# The *higBA* Toxin-Antitoxin Module From the Opportunistic Pathogen *Acinetobacter baumannii* – Regulation, Activity, and Evolution

**DOI:** 10.3389/fmicb.2018.00732

**Published:** 2018-04-12

**Authors:** Julija Armalytė, Dukas Jurėnas, Renatas Krasauskas, Albinas Čepauskas, Edita Sužiedėlienė

**Affiliations:** ^1^Institute of Biosciences, Life Sciences Center, Vilnius University, Vilnius, Lithuania; ^2^Cellular and Molecular Microbiology, Department of Molecular Biology, Université Libre de Bruxelles, Brussels, Belgium

**Keywords:** toxin-antitoxin, HigBA, plasmid maintenance, *Acinetobacter baumannii*, protein complex

## Abstract

*Acinetobacter baumannii* is one of the major causes of hard to treat multidrug-resistant hospital infections. *A. baumannii* features contributing to its spread and persistence in clinical environment are only beginning to be explored. Bacterial toxin-antitoxin (TA) systems are genetic loci shown to be involved in plasmid maintenance and proposed to function as components of stress response networks. Here we present a thorough characterization of type II system of *A. baumannii*, which is the most ubiquitous TA module present in *A. baumannii* plasmids. *higBA* of *A. baumannii* is a reverse TA (the toxin gene is the first in the operon) and shows little homology to other TA systems of RelE superfamily. It is represented by two variants, which both are functional albeit exhibit strong difference in sequence conservation. The *higBA2* operon is found on ubiquitous 11 Kb pAB120 plasmid, conferring carbapenem resistance to clinical *A. baumannii* isolates and represents a *higBA* variant that can be found with multiple sequence variations. We show here that *higBA2* is capable to confer maintenance of unstable plasmid in *Acinetobacter* species. HigB2 toxin functions as a ribonuclease and its activity is neutralized by HigA2 antitoxin through formation of an unusually large heterooligomeric complex. Based on the *in vivo* expression analysis of *gfp* reporter gene we propose that HigA2 antitoxin and HigBA2 protein complex bind the *higBA2* promoter region to downregulate its transcription. We also demonstrate that *higBA2* is a stress responsive locus, whose transcription changes in conditions encountered by *A. baumannii* in clinical environment and within the host. We show elevated expression of *higBA2* during stationary phase, under iron deficiency and downregulated expression after antibiotic (rifampicin) treatment.

## Introduction

*Acinetobacter baumannii* is an emerging Gram-negative opportunistic pathogen, causing serious hospital-acquired infections (Antunes et al., 2014). These bacteria are well adapted to survive in hospital environment such as intensive care units, burn wards, and field hospitals (Rosa et al., 2014). During the last decade, several highly successful multidrug-resistant *A. baumannii* clonal lineages have spread in clinical settings worldwide causing difficult to treat hospital outbreaks (Howard et al., 2012). *A. baumannii* is known for its ability to withstand harsh environmental conditions such as prolonged periods of dryness, disinfectants, and antibiotic treatment (Jawad et al., 1998; Cardoso et al., 2010; Nwugo et al., 2012).

Bacterial type II toxin-antitoxin (TA) systems are the most ubiquitous among six types of prokaryotic toxin-antitoxin systems (TAs), known to date (Chan et al., 2016; Page and Peti, 2016; Rocker and Meinhart, 2016). They are encoded on the low copy plasmids or chromosomes and code for two proteins, one of which (toxin) is toxic to the cell, whereas the other (antitoxin) neutralizes its toxicity by forming strong protein–protein complex, which is non-harmful. Upon release from the complex, the toxin acts within the cell by interfering with essential processes, such as protein (Díaz-Orejas et al., 2010; Goeders and Van Melderen, 2014) or DNA synthesis (Harms et al., 2015), cell wall synthesis (Mutschler et al., 2011), and cell division (Masuda et al., 2012). The toxin action results in a rapid cell growth arrest or even leads to cell death (Page and Peti, 2016). The majority of toxins from type II systems, characterized to date, are endoribonucleases (Cook et al., 2013), which, if not neutralized by its cognate antitoxin protein, cleave mRNAs at specific sequences either within or outside the ribosome and inhibit translation (Chan et al., 2016). The plasmid-borne type II TAs often function as plasmid stabilization elements by allowing growth of the cells that inherit plasmid with the TA system upon segregation, whereas cells that have lost plasmid are killed by more stable toxin after the more labile antitoxin is degraded by proteases (Engelberg-Kulka and Glaser, 1999; Hernández-Arriaga et al., 2015). The biological role of chromosomally encoded type II systems is not clearly elucidated yet. The proposed functions of type II TAs range from viewing them as selfish DNA, anti-addiction elements to stress-responsive genes, which can regulate bacterial growth and survival adapting to various environmental changes (Magnuson, 2007; Van Melderen and Saavedra De Bast, 2009; Ramisetty and Santhosh, 2017). The TA systems can adjust the metabolic processes at a large scale, such as shutting down protein synthesis and switching to a dormant cellular state (Kędzierska and Hayes, 2016; Lee and Lee, 2016).

Genome analysis has shown a wide variety of TA modules in pathogenic species (Makarova et al., 2009; Leplae et al., 2011). The role of TAs in the life of bacterial pathogens is now beginning to be explored (Fernández-García et al., 2016; Kędzierska and Hayes, 2016; Lee and Lee, 2016; Lobato-Márquez et al., 2016a). Recent reports have demonstrated the significance of TAs in the stabilization of virulence plasmids in *Shigella* (McVicker and Tang, 2016) and *Salmonella enterica* (Lobato-Márquez et al., 2016b), superintegron in *Vibrio cholerae* (Iqbal et al., 2015), also in the promoting of *S. enterica* persister formation (Cheverton et al., 2016; Jaiswal et al., 2016) and in mediating the transcriptional response to environmental cues in *Helicobacter pylori* and *Brucella abortus* (Heaton et al., 2012; Cárdenas-Mondragón et al., 2016).

We have recently shown that type II HigBA TA system is one of the most prevalent plasmid-borne TA systems in *A. baumannii* isolates of clinical origin. It is encoded by the *higBA*_Ab_ operon, where *higB*_Ab_ toxin gene (357 bp) precedes *higA*_Ab_ antitoxin (303 bp) (Jurenaite et al., 2013; Sužiedėlienė et al., 2016). *higBA*_Ab_ locus was also found to be encoded by the newly observed ubiquitous *A. baumannii* 11 kb plasmid pAB120. Plasmid carries two copies of *bla*_OXA-72_ genes, conferring resistance to carbapenems, a broad spectrum β-lactam antibiotics class, which is used to treat *A. baumannii* infections (Povilonis et al., 2013; Supplementary Figure S1). Here we report characterization of *A. baumannii* type II TA system, by demonstrating that *higBA* locus is represented in *A. baumannii* by two functional variants, named *higBA1*_Ab_ and *higBA2*_Ab_. The *higBA2*_Ab_ is encoded on pAB120 plasmid and was further thoroughly characterized. We demonstrate that HigB2_Ab_ toxin acts as a ribonuclease and forms an unusually large complex with the antitoxin. Both HigA2_Ab_ and the HigBA2_Ab_ protein complex transcriptionally autoregulate their own operon. We show that *higBA2*_Ab_ represents a stress responsive TA locus, which also possesses plasmid stabilization ability.

## Materials and Methods

### Bacterial Strains and Growth Conditions

*Escherichia coli* and *Acinetobacter* strains were grown in LB at 37°C with appropriate antibiotics added (ampicillin 100 μg/mL, meropenem 8 μg/mL, gentamicin 10 μg/mL, kanamycin 50 μg/mL, and chloramphenicol 30 μg/mL), unless otherwise indicated. The strains and plasmids used in the study are described in Supplementary Table S1. *A. baumannii* was transformed with plasmid pAB120 variants by electroporation, and selected on LB containing meropenem. Minimal inhibitory concentration (MIC) values were detected as described (Wiegand et al., 2008).

### Plasmid Construction

Gene cloning procedures were performed using high-fidelity Phusion polymerase (Thermo Fisher Scientific), cleavage with restriction enzymes and ligation was performed according manufacturers recommendations (New England Biolabs, Thermo Fisher Scientific). All final constructs were verified by sequencing. pAB120ΔhigBA: to introduce *higBA2*_Ab_ deletion to pAB120, primers described in Supplementary Table S2 were used for inverse PCR (Imai et al., 1991). The resulting PCR product was cleaved with NcoI and ligated. The deletion of *higBA2*_Ab_ was confirmed by PCR. pAcORI^∗^higBA: a derivative of the plasmid pWH1266 (Hunger et al., 1990; Supplementary Table S1), exhibiting faulty inheritance in *Acinetobacter* sp. was constructed. pAcORI^∗^ plasmid contained pUC19 backbone with introduced gentamicin resistance gene and a defective *Acinetobacter calcoaceticus* ORI from pWH1266, amplified using primers listed in Supplementary Table S2. *higBA2*_Ab_ TA system from pAB120, together with predicted promoter region, was then cloned to pAcORI^∗^ using primers in Supplementary Table S2. BACTH constructs: for T18 and T25 fusions plasmids pUT18 and pKNT25 (Euromedex BACTH System Kit) were used. *higB2*_Ab_, *higA2*_Ab_, *relB*_Ab_, *relE*_Ab_ genes were PCR-amplified using primer pairs described in Supplementary Table S2. pAB120 plasmid was used as a template for *higBA2*_Ab_ gene amplification, while for *relBE*_Ab_ *A. baumannii* clinical (35, ECII) strain was used (Jurenaite et al., 2013). Both toxins and antitoxins were N-terminally fused to appropriate Cya domains. Protein expression plasmids: for protein purification, *higBA2*_Ab_ operon was cloned to pET28b protein expression vector, fusing the antitoxin (C-terminally) or the toxin (N-terminally) with his-tag (Supplementary Table S2). To construct plasmids containing TEV cleavage sites, the thrombin recognition site in the plasmid was replaced to TEV site by inverse PCR using primers listed in Supplementary Table S2 to generate pET-His6-TEV-HigB-HigA plasmid. For pET-HigB-His6-TEV-HigA, TEV site and His-tag was first removed from previous plasmid and then added to the N-terminus of *higA2*_Ab_. Constructs for promoter repression assay: pPROBE’-gfp vectors were constructed by inserting PCR amplified predicted promoter DNA sequences (200 bp in length upstream of *higB2*_Ab_ or *higA2*_Ab_ genes; primers indicated in Supplementary Table S2) upstream of *gfp* gene. *higBA2*_Ab_ and *higA2*_Ab_ genes were cloned to pBAD24 under arabinose inducible promoter (primers indicated in Supplementary Table S2). pBAD-higBpAB120 and pUHEcat-higApAB120 for kill-rescue assay: the plasmids were constructed as described elsewhere (Jurenaite et al., 2013).

### Bacterial Adenylate Cyclase Two Hybrid System (BACTH) Assay

Five independent clones, with compatible toxin and antitoxin containing pKNT25 and pUT18 vectors were grown in LB for 16 h, then the cultures of the five clones were mixed and 5 μL of the mix was spotted on LB agar plates containing appropriate antibiotics, 100 μg/mL IPTG and 100 μg/mL X-Gal. The plates were incubated for 24 h at 30°C.

### Purification of HigBA2_Ab_ Protein Complex

For purification of His-HigBA2 and HigBA2-His protein complexes, plasmids pET-His-HigBA or pET-HigBA-His, were introduced into *E. coli* strain BL21 (DE3) (Supplementary Table S1) and the expression of protein complex induced by incubation with 1 mM IPTG for 4 h during mid-logarithmic phase. Cells were collected by centrifugation for 10 min at 4°C 5500 g, bacterial pellets were resuspended in lysis buffer (20 mM NaH_2_PO_4_ pH 7.4, 500 mM NaCl, 20 mM imidazole) and lysed by sonication. Lysate was centrifuged for 10 min at 4°C 13000 g to remove cell debris. The protein complexes were purified from soluble fraction by affinity chromatography, using 1 mL HisTrapHP^TM^ nickel-Sepharose column (GE Healthcare), equilibrated with 20 mM NaH_2_PO_4_ pH 7.4, 500 mM NaCl, 20 mM imidazole buffer. After loading the protein lysate, the column was washed with the same buffer for column volumes followed by 10 volumes of wash buffer (20 mM NaH_2_PO_4_ pH 7.4, 500 mM NaCl, 50 mM imidazole) to remove impurities. Proteins were eluted by linear gradient using buffer 20 mM NaH_2_PO_4_ pH 7.4, 500 mM NaCl, 500 mM imidazole. The eluted fractions were desalted using Sephadex G–25 (GE Healthcare) column, exchanging the buffer to 20 mM NaH_2_PO_4_ pH 7.2, 300 mM NaCl. Eluted proteins were analyzed by 15% sodium dodecyl sulfate-polyacrylamide gel electrophoresis (SDS–PAGE), stained with Coomassie Brilliant Blue. To generate tag-less complex, His-TEV-HigBA2 proteins were incubated with TEV protease at a molar ratio of 1 to 100 overnight at room temperature.

### Protein Complex Size Determination by Size-Exclusion Chromatography

Purified His-HigBA2 and HigBA2-His complexes were analyzed by FPLC gel filtration chromatography using Superose 12 10/300 GL column (GE Healthcare). Column was washed with 20 mM NaH_2_PO_4_ pH 7.2, 300 mM NaCl buffer. Gel filtration flow rate was 0.5 mL/min. The relative quantities of the proteins present in the fractions were determined after SDS–PAGE using Image Lab software (Bio-Rad). The tag-less HigBA2 complex was analyzed by Superdex 200 16/60 column (GE Healthcare), using 50 mM Tris, pH 8.0, 500 mM NaCl buffer. The protein molecular mass standards were thyroglobulin (670 kDa); γ-globulin (158 kDa); bovine serum albumin (66 kDa); ovalbumin (43 kDa); myoglobin (17 kDa); vitamin B12 (1.35 kDa) (Bio-Rad Gel filtration standard 151–1901) and Blue Dextran (GE Healthcare).

### Toxin Purification From Protein Complex

To separate HigB2 toxin from His-HigBA2 complex, denaturant-induced dissociation of the toxin–antitoxin complex on-column method was used (Sterckx et al., 2015). Briefly, the protein complex was denatured on the affinity column using guanidine HCl buffer (5 volumes of 50 mM Tris, pH 8.0, 500 mM NaCl, 5 M guanidine HCl), followed by renaturing on the resin (5 volumes of 25 mM Tris pH 8.0, 250 mM NaCl, 5% glycerol, followed by 5 volumes of 25 mM Tris pH 8.0, 250 mM NaCl, 1% glycerol). The toxin was eluted with 50 mM Tris pH 8.0, 500 mM NaCl, 200 mM imidazole, then immediately dialyzed to 50 mM Tris–HCl pH 8.0.

### RNA Cleavage Analysis *in Vitro*

Total *A. baumannii* RNA, 5S rRNA and *E. coli* total tRNA were used as substrates for *in vitro* RNA cleavage analysis by HigB2 toxin. The mixtures (20 μL) contained 1.5 μg total RNA or 3 μg of 5S or tRNA in 25 mM Tris–HCl pH 7.8, and 0–1 μM final concentration of His-HigB2 protein. As a control, 1 μM of His-HigBA2 protein complex was added. The mixes were incubated for 30 min at 37°C and fractionated using 2% agarose electrophoresis.

### Promoter Activity and Repression Measurements *in Vivo*

For promoter activity assays DJ624Δ*ara* cells were transformed with pPROBE’-gfp vectors with or without inserted predicted promoter sequences upstream from *gfp*. Together, pBAD24, pBAD24-HigBA or pBAD24-HigA vectors were co-transformed. Overnight cultures of resulting strains were diluted to optical density at 600 nm (OD_600_) = 0.02 in minimal M9 media with casamino acids, 50 μg/mL kanamycin, 100 μg/mL ampicillin and 0.2% glucose. 1 mL of cultures was grown in glass bottom black 24 well plates (Greiner) in Spectramax microplate reader at 37°C with constant shaking. OD_600_ and fluorescence (excitation 485 nm emission 520 nm) were registered every 15 min. After 3 h of growth 0.2% arabinose was added to induce the protein expression from pBAD24 vectors and measurements were continued for 10 more hours.

### Analysis of Gene Expression by qPCR

*Acinetobacter baumannii* clinical strain K60 (Povilonis et al., 2013), containing pAB120 plasmid, was grown in LB, to exponential phase (OD_600_ = 1) and to stationary phase (48 h, OD_600_ = 4.5). For stress conditions, the cells were grown in LB with 200 mM 2,20-bipyridine (Eijkelkamp et al., 2011) or in LB with 1 or 2% ethanol to OD_600_ = 1. For antibiotic stress, 1/10 of MIC values for gentamicin and rifampicin (2 and 0.3 μg/mL, respectively) and 1/2 MIC for meropenem (16 μg/mL) was added to exponentially growing cells and incubated for 1 h to a final OD_600_ = 1. Total RNA was isolated, DNA removed and cDNA synthesized as recommended by the kit supplier (Thermo Fisher Scientific). qPCR was performed using primer pairs listed in Supplementary Table S2 (all primers exhibited 100–103% amplification efficiency at selected concentrations). The changes in gene expression were calculated as ΔΔCt, using *rpoB* as house-keeping gene. At least three biological replicas were performed.

### Plasmid Stability Assay

Bacteria containing plasmid of interest were grown in LB containing appropriate antibiotic as an overnight culture (16 h), and then diluted 500-fold to a fresh media without antibiotic. The culture was restarted to a new batch of LB without antibiotic every 12 h. After each inoculation the colony forming units (CFU) of antibiotic resistant (retaining the plasmid encoding resistance) and sensitive bacteria (plasmid lost) was calculated by serial dilutions and plating on LB agar with or without appropriate antibiotic, and plasmid retention was calculated as a percentage of resistant bacteria.

### Plasmid Copy Number (PCN) Calculation

Plasmid copy number (PCN) was calculated as described elsewhere (Providenti et al., 2006). Briefly, the bacteria were grown for 24 h without antibiotic pressure, the cells were collected and their lysates used for qPCR, with primers detecting *rep* gene of the pAB120 plasmid and *rpoB* as genome encoded control. Copy number was calculated as PCN = E^ΔCt^ (*E* = 2, if the product amplifies at 100% efficiency, which was the case for the primers used). The experiment was independently repeated 10 times.

### Kill-Rescue Assay

The assay was performed as previously described (Jurenaite et al., 2013). Briefly, *E. coli* BW25113 F‘ pUHEcat-“antitoxin” pBAD-“toxin” strains were grown in LB medium until the early logarithmic phase (optical density at 600 nm (OD_600_) = 0.1). The culture was then induced with arabinose (0 or 0.02%) and/or IPTG (0, 0.1, and 1 mM). The growth was observed in Tecan Infinite M200 Pro plate reader at 37°C with shaking.

### *In Silico* Analysis

Cluster of GP49-domain toxin family was prepared as neighbor joining tree (Saitou and Nei, 1987) using BLOSUM62 matrix, average distance tree of two major lineages of HigB toxins based on sequence identity as well as alignment of HigB_Ab_ toxins obtained using ClustalW and Jalview 2.10.0b1.

## Results

### *higBA*_Ab_ System in *A. baumannii* Is Represented by Two Variants

**FIGURE 1:**
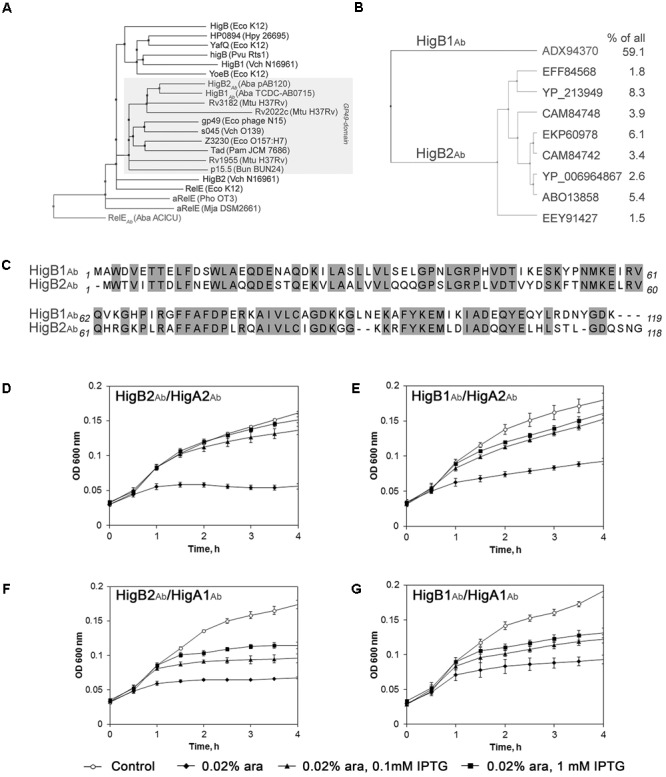
Phylogenetic and homology analysis of *Acinetobacter baumannii* HigB_Ab_. **(A)** Cluster of GP49-domain toxin family within RelE superfamily, represented as neighbor joining tree. Gray box covers all GP49-domain proteins. The full organism names are provided in the Supplementary Material (Supplementary Table S3); **(B)** Two major lineages of HigB_Ab_ toxins, represented as average distance tree. All proteins that have more than 1% identical hits in all deposited sequences are shown and their frequency in percentage is provided; **(C)** Alignment of pAB120-borne HigB2_Ab_ (Povilonis et al., 2013) and HigB1_Ab_ (Jurenaite et al., 2013) toxin sequences. Identical amino acid residues are outlined in gray boxes. All alignments were prepared as described in Section “Materials and Methods”; **(D–G)** Growth of *Escherichia coli* BW25113F′ harboring components of *higBA*_Ab_ and their combinations. The *E. coli* strains with pUHE-“antitoxin” and pBAD30-“toxin” plasmids were grown until OD_600_ = 0.1 and induced with 0.02% arabinose and/or with 0.1–1 mM IPTG as described in Section “Materials and Methods.” The data from three independent experiments are represented, the error bars indicate standard deviation.

*Acinetobacter baumannii higBA* module has been classified on the basis of low but significant homology of its predicted GP49-like domain toxin HigB to the RelE/ParE superfamily toxins (Jurenaite et al., 2013). Further homology search has shown that the closest GP49-domain homologues of HigB_Ab_ mentioned in the literature are *Mycobacterium tuberculosis* Rv2022c-Rv2021c (also called *higBA2*) and Rv3182-Rv3183 (*higBA3*) TA systems, whose toxins share more than 40% protein sequence identity with HigB_Ab_ (**Figure 1A**). Based on the BLAST search, close homologues of HigB_Ab_ (more than 50% sequence identity) are present in other gammaproteobacteria closely related to *Acinetobacter*, such as *Psychrobacter*, as well as various members of *Enterobacteriaceae* family, such as *Klebsiella* (Supplementary Figure S2).

Strikingly, BLAST search revealed a variety of amino acid sequences for HigB_Ab_ toxin in *A. baumannii* species. Therefore, we have aligned all *A. baumannii* HigB_Ab_ sequences existing to date and found that they are, in fact, represented by two distinct versions of HigB_Ab_ modules showing around 60% sequence identity (**Figures 1B,C**). The most prevalent version is largely conserved (called HigB1_Ab_ further), while the other version of HigB_Ab_ is represented by many sequence variations ranging from 85 to 99% identity (HigB2_Ab_) (**Figure 1B**). The *higBA*_Ab_ module found in plasmid pAB120 (Povilonis et al., 2013), which we characterize in this study, represents the less conservative version *higBA2*_Ab_, while the conserved version *higBA1*_Ab_ has been characterized previously as a representative *higBA*_Ab_ module carried by strain 35 in a pilot study of *A. baumannii* TA systems (Jurenaite et al., 2013). We were interested whether the two versions of TA systems constitute stand-alone TA modules. Expression of both versions of HigB_Ab_ toxin resulted in an inhibition of *E. coli* growth, and interestingly, co-expression of HigA_Ab_ antitoxin counteracted toxin-mediated growth inhibition regardless the HigA_Ab_ variant used for growth rescue (**Figures 1D–G**). This experiment has shown that the components of two TA variants are still able to interact despite 42 and 45% amino acid sequence difference between the toxins and antitoxins of the two groups, respectively. Structure models of both versions of HigB and HigA (Supplementary Figure S3) also predicted high similarity of folds, despite some differences at the termini of the proteins. Notably, the pAB120-borne HigBA2_Ab_ pair exhibited more pronounced kill-rescue effect compared to the more conserved HigBA1_Ab_ version (**Figures 1D** vs. **G**). These results are in line with the proposed anti-addiction hypothesis (Saavedra De Bast et al., 2008), where the conserved but less active HigBA1_Ab_ module could provide evolutionary pressure for the HigBA2_Ab_ lineage.

We then searched for *Acinetobacter* sp. strains having both *higBA*_Ab_ versions in the GenBank sequence database. We were not able to find two distinct versions of *higBA*_Ab_ on the same plasmid or in the same strain. This phenomenon has been reported previously by analysis of identity of bacterial and chromosomal TAs (Ramisetty and Santhosh, 2016). However, we were able to find two sequenced *Acinetobacter* sp. isolates with copies of *higBA*_Ab_ on several plasmids [isolates DUT-2 (GenBank accessions CP014652, CP014654, CP014655) and VE-C3 (GenBank accessions NC_010310, NZ_ALIG01000010)], and five plasmids containing two copies of same version of *higBA*_Ab_ (GenBank accessions CM008888, CP010369, CM001803, NC_010404, NC_025173) (Supplementary Table S3). Interestingly, all of them had the less conserved *higBA2*_Ab_ versions. In two cases [plasmid “unnamed2” from *A. baumannii* strain ZQ1 (Supplementary Table S3) and DUT-2 (plasmids CP014652, CP014654)], both copies of *higBA*_Ab_ were identical, indicating a recent event of duplication. The other cases with two distinct *higBA2*_Ab_ variants had 86 to 88% DNA sequence identity. The presence of two diverse copies of *higBA*_Ab_ in the same bacteria or on a single plasmid could indicate the event of ongoing evolution of the system and its separation into two different TA systems.

### HigB2_Ab_ and HigA2_Ab_ Proteins Form Complex

**FIGURE 2:**
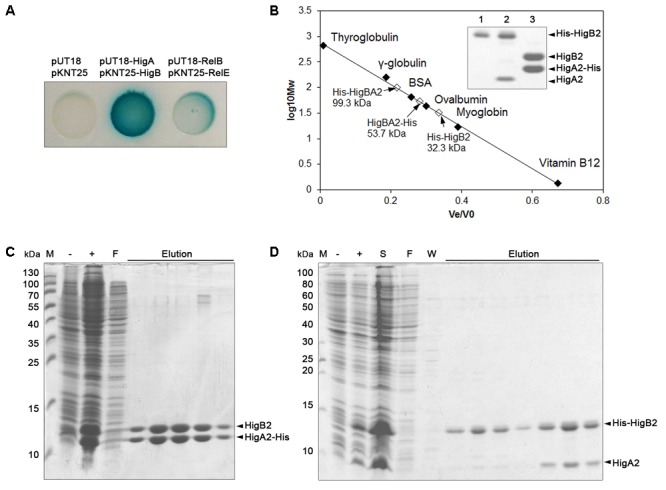
Analysis of HigB2_Ab_–HigA2_Ab_ protein interaction. **(A)** Two-hybrid analysis of interaction of toxin-antitoxin (TA) components. *E. coli* BTH101 with two plasmids, one encoding Cya-T18 fused to *A. baumanii* antitoxins HigA2_Ab_ or RelB_Ab_, and another encoding Cya-T25 fused to toxins HigB2_Ab_ or RelE_Ab_. The ability of the proteins to interact and reconstitute functional Cya from T25 and T18 was observed as blue colony formation when grown on LB agar with IPTG and X-gal for 24 h; **(B)** Size-exclusion chromatography and SDS–PAGE analysis of His-HigBA2 and HigBA2-His protein complexes. The proteins used for molecular mass standard curve are indicated as black diamonds, empty diamonds indicate the positions of His-HigBA2 and HigBA2-His protein complexes and single His-HigB2 protein and their calculated size; inside box – 15% SDS–PAGE gel analysis lane 1 – His-HigB2, lane 2 – His-HigBA2, lane 3 – HigBA2-His proteins and complexes after gel filtration; **(C,D)** *E. coli* BL21(DE3) strain, containing pET-HigBA-His **(C)** and pET-His-HigBA **(D)** plasmids were grown to mid-exponential phase and protein expression was induced with 1 mM IPTG for 4 h. Cells were disrupted by sonication and the His-tag containing proteins were purified by affinity chromatography from the soluble fraction as described in Section “Materials and Methods”. Proteins were visualized by 15% SDS–PAGE stained with Coomassie Brilliant Blue. M – protein molecular mass markers (band sizes in kDa are shown on the Left), –/+ cell lysate before and after induction with IPTG, S – soluble protein fraction after cell disruption, F – protein purification flow-through fraction, W – protein purification wash fraction, Elution – protein purification elution fractions.

pAB120 plasmid-borne HigBA2_Ab_ and its toxin HigB2_Ab_ shares only low similarity to RelE/ParE type TA toxins. Therefore we asked whether this TA system is and acts as a canonical one. Type II TA systems are known to employ different mechanisms of action and their proteins may form different oligomeric complexes (Chan et al., 2016; Rocker and Meinhart, 2016). We asked whether HigB2_Ab_ and HigA2_Ab_ form a physical complex which neutralizes toxicity in a typical way to type II TA systems. For this purpose we employed bacterial adenylate cyclase two hybrid system (BACTH) (Karimova et al., 1998) and verified that HigB2_Ab_ toxin and HigA2_Ab_ antitoxin directly interact *in vivo* by restoring adenylate cyclase activity (**Figure 2A**). Moreover, the *A. baumannii* RelE_Ab_ and RelB_Ab_ proteins [homologues of well-described RelBE TA system of *E. coli* (Jurenaite et al., 2013)], which we used as a control, showed even weaker interaction in comparison to that of HigB2_Ab_ and HigA2_Ab_.

To further assess the complex forming ability between *A. baumannii* HigB2_Ab_ and HigA2_Ab_ proteins, we have constructed two versions of the expression vectors encoding *higBA2*_Ab_ operon with HigB2_Ab_ toxin N-terminal His-tag fusion (pET-His-HigBA) and with antitoxin HigA2_Ab_ C-terminal His-tag fusion (pET-HigBA-His). Proteins were expressed in *E. coli* and purified by affinity chromatography as described in Section “Materials and Methods.” In both cases HigB2_Ab_ and HigA2_Ab_ proteins co-purified, indicating that they form a strong complex (**Figures 2C,D**). To assess the size of complexes we have analyzed them by size-exclusion chromatography. The elution profiles showed single peaks (not shown) corresponding to the entities with estimated molecular masses of approximately 99.3 kDa and 53.7 kDa for His-HigBA2_Ab_ and HigBA2_Ab_-His complexes, respectively (**Figure 2B**). Protein analysis of pooled fractions that corresponded eluted peaks by 15% SDS–PAGE and subsequent gel densitometric analysis showed two bands present in ratio (toxin:antitoxin) of approximately 1.5:1 and 1:1 (**Figure 2B**). As the differences in observed complex sizes could have been due to the interference of His-tag, we aimed to confirm the complex size with the tag-less protein complex. To eliminate the probable His-tag effect, we constructed plasmids with cleavable His-tags situated at the N-terminus of HigB2_Ab_ (plasmid pET-His6-TEV-HigB-HigA) or HigA2_Ab_ (plasmid pET-HigB-His6-TEV-HigA). However, the cleavage of His-tag was only efficient for the protein complex with N-terminal His-tag fusion of HigA2_Ab_. Upon removal of His-tag from HigBA2_Ab_ complex, size exclusion chromatography resulted in an entity of approximately 109 kDa (Supplementary Figure S4), thus confirming that HigBA2_Ab_ is able to form unusually large complex.

### HigB2_Ab_ Toxin Is a Ribonuclease

**FIGURE 3:**
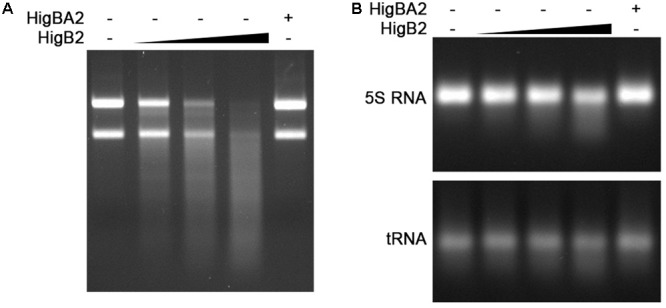
HigB2_Ab_ toxin acts as a ribonuclease. His-HigBA2 protein complex at the concentration of 1 μM and His-HigB2 toxin at the concentrations of 0, 0.25, 0.5, and 1 μM, were incubated with 1.5 μg of total *A. baumannii* RNA **(A)** and with 3 μg of *E. coli* 5S rRNA **(B)** in 10 μl reactions for 30 min at 37°C. The samples were visualized in 1% agarose gel.

We have demonstrated previously, that HigB1_Ab_ toxin from *higBA1*_Ab_ system (named as *higBA*_Ab_ in the previous research) when expressed in *E. coli* caused degradation of cellular RNAs and inhibited translation by impairing the incorporation of protein synthesis precursor, therefore suggesting that it might act as a ribonuclease (Jurenaite et al., 2013). To investigate whether HigB2_Ab_ possesses a ribonuclease activity *in vitro*, we have purified recombinant His-HigB2 toxin from His-HigBA2_Ab_ complex and tested whether it is able to degrade *A. baumannii* total RNA as well as RNA with known secondary structure such as *E. coli* 5S rRNA and tRNA. HigB2_Ab_ toxin efficiently degraded total *A. baumannii* RNA in a dose dependent manner, whereas in a complex with HigA2_Ab_ protein its ribonuclease activity was blocked (**Figure 3A**). The degrading activity against *E. coli* 5S rRNA was only detected using high concentrations of HigB2_Ab_ toxin (**Figure 3B**), whereas effect against tRNA at the same conditions was negligible, indicating that HigB2_Ab_ toxin more likely targets unstructured RNAs.

### HigA2_Ab_ Antitoxin Represses Transcription From *higBA2*_Ab_ Promoter Whereas HigB2_Ab_ Toxin Acts as a Corepressor

**FIGURE 4:**
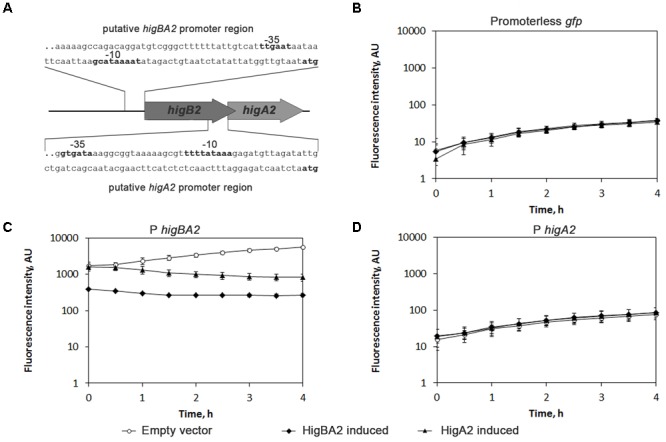
Analysis of HigBA2_Ab_ interaction with own promoter region. **(A)** Schematic representation of *A. baumannii higBA2*_Ab_ locus. A part of pAB120 plasmid containing *higBA2_Ab_* operon with predicted promoter is presented (not to scale). Predicted promoter DNA sequences (100 bp out of 200 bp cloned to vectors) used for promoter activity assay are shown, predicted –35 and –10 regions (BPROM) and ATG are indicated in bold; **(B–D)** The assessment of activity of predicted *higBA2*_Ab_ operon promoters. Predicted promoter regions of *higBA2*_Ab_ operon and *higA2*_Ab_ gene were cloned into pPROBE vector, placing them upstream the *gfp* gene as described in Section “Materials and Methods”. Then pPROBE with respective promoter sequence or lacking any promoter (Promoterless *gfp*) was co-transformed into *E. coli* DJ624Δ*ara* harboring pBAD24 plasmids, with cloned *higBA2*_Ab_ operon or *higA*_Ab_ antitoxin genes under arabinose inducible promoter. After addition of 0.2% arabinose, GFP fluorescence was measured every 30 min, reflecting the effect of induced proteins on the activity of measured promoters. Three independent experiments were performed, error bars indicate standard deviation. AU, arbitrary units.

Type II TA complexes or single antitoxin proteins have been shown to bind to the operator DNA of their own promoter and negatively autoregulate operon transcription (Kedzierska et al., 2007). HigA2_Ab_ has a HTH domain and could bind to DNA alone or in complex with HigB2_Ab_. It is not known how reverse TA systems, where the toxin is the first gene in the operon, regulate their toxin:antitoxin ratio. We looked if an additional promoter could be found for *higA*_Ab_, indicating additional regulation for antitoxin expression. BPROM tool (Solovyev and Salamov, 2011) predicted promoter elements (-10 and -35) upstream the *higBA2*_Ab_ operon and *higA2*_Ab_ coding sequence (**Figure 4A**), indicating that besides promoter for the operon, *higA2*_Ab_ might also have an additional promoter. We have tested the activity of these promoters and their regulation by HigBA2_Ab_ complex and HigA2_Ab_ protein. 200 bp DNA fragments containing *higBA2*_Ab_ and *higA2*_Ab_ promoters were cloned upstream of the *gfp* gene (plasmids pPROBE-P*higBA*-gfp and pPROBE-P*higA*-gfp). Promoter activities were measured by following GFP fluorescence upon induction of HigA2_Ab_ antitoxin and HigBA2_Ab_ complex (plasmids pBAD24-HigA, pBAD24-HigBA) in *E. coli*. Promoterless *gfp* (plasmid pPROBE-gfp) was used to assess autofluorescence level of the cells. Induction of HigA2_Ab_ expression strongly repressed the activity of *higBA2*_Ab_ promoter, and the induction of HigBA2_Ab_ complex showed even more pronounced inhibitory effect (**Figure 4C**). Putative *higA2*_Ab_ promoter located within the coding sequence of *higB2*_Ab_ gene did not display any activity (**Figures 4B,D**). Our results clearly show that *higBA2*_Ab_ operon is transcriptionally autorepressed by its cognate antitoxin HigA2_Ab_, and HigB2_Ab_ toxin acts as a co-repressor. The predicted *higA2*_Ab_ promoter was not functional.

### Expression of *A. baumannii higB2*_Ab_ and *higA2*_Ab_ Genes in Stress Conditions

**FIGURE 5:**
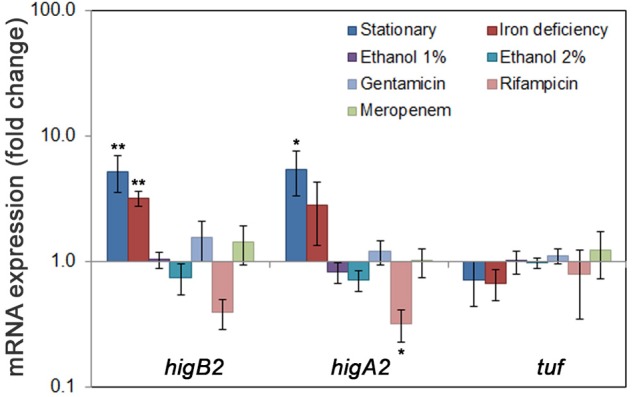
Expression of *A. baumannii higBA2_Ab_* locus during stress conditions. *A. baumannii* clinical strain K60 was grown in LB to exponential phase (OD_600_ = 1) and to stationary phase (blue bars); in conditions mimicking iron deficiency (LB with or without 2,2′-bipyridine) (red bars), in LB with 1 or 2% ethanol added (purple and cyan bars, respectively) and LB with antibiotics (gentamicin, rifampicin, meropenem, in light blue, light red, and light green, respectively) added. The bacteria were collected at OD_600_ = 1, except for stationary phase, where the bacteria were grown for 48 h reaching OD_600_ of 4.5. Total RNA was isolated and RT-qPCR performed to detect the expression of TA genes as described in Section “Materials and Methods”. The differences in transcript amounts were evaluated by comparative Ct method (ΔΔCt) using *rpoB* as a house-keeping gene. At least three biological replicas were performed, error bars indicate standard deviation. Statistically significant difference from *tuf* expression, used as a control, is indicated by one asterisk (*t*-test, *p* < 0.05), or two asterisks (*t*-test, *p* < 0.005).

We have demonstrated that HigB2_Ab_ and HigA2_Ab_ form a strong complex which causes repression of its own operon. We next asked how *higBA2*_Ab_ locus is expressed in *A. baumannii* K60 (Povilonis et al., 2013) under various conditions. We have chosen to investigate growth and stress conditions relevant to those found in clinical environment and within the host: the stationary phase, conditions mimicking the iron deficiency, growth with ethanol and sub-lethal amounts of antibiotics. The expression changes in both *higB2*_Ab_ and *higA2*_Ab_ were evaluated separately by qPCR to see if there are any differences in expression levels which would indicate the presence of separate transcripts. We have found that transcripts of *higB2*_Ab_ and *higA2*_Ab_ genes were more abundant in stationary phase (more than fivefold increase) comparing to the exponential growth conditions (**Figure 5**). Interestingly, the iron deficiency caused by the addition of iron chelator 2,2′-bipyridine (Eijkelkamp et al., 2011) to the LB medium resulted in the up to threefold induction of the *higB2*_Ab_ and *higA2*_Ab_ genes (**Figure 5**). The presence of ethanol in the media did not have pronounced effect on the gene expression (**Figure 5**). We further tested sub-lethal concentrations of antibiotics. As *A. baumannii* strain K60 was highly resistant to several classes of antibiotics [aminoglycosides, β-lactams, fluoroquinolones, (Supplementary Table S4)], we chose antibiotics which exhibited moderate to low MIC (gentamicin MIC 20 μg/mL, rifampicin MIC 3.125 μg/mL). The antibiotics were added at 1/10 of the MIC, as higher concentrations resulted in severe growth impairment. Effect of meropenem was also tested (1/2 of MIC), due to the presence *bla_OXA-72_* gene in the proximity to *higBA2*_Ab_ in pAB120 plasmid. No significant changes in *higBA2*_Ab_ expression were observed for gentamicin or meropenem, but the addition of rifampicin caused a decrease of expression of both genes (**Figure 5**). We therefore conclude that *higBA2_Ab_* module could be expressed during stress conditions linked to stationary phase and iron deficiency stress, as well as it could play a role during RNA synthesis inhibition or yet in other unknown conditions. Additionally, since all expression changes were similar for both *higB2*_Ab_ and *higA2*_Ab_, we can expect both genes to be expressed from a single transcript of the whole operon.

### The Role of *higBA2*_Ab_ TA System in Stabilization of *Acinetobacter* Plasmids

**FIGURE 6:**
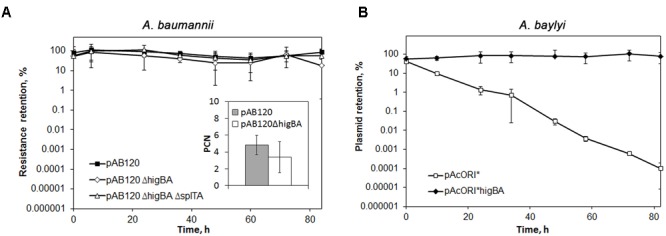
The role of *A. baumannii higBA2*_Ab_ locus in plasmid stabilization. Bacteria containing plasmids were grown in LB without antibiotic pressure, restarting the culture every 12 h. The colony forming units (CFU) of total bacteria and antibiotic resistant bacteria was assessed by serial dilution and plating. At least three independent experiments were performed, error bars indicate standard deviation. **(A)** The effect of *higBA2*_Ab_ deletion on *A. baumannii* pAB120 plasmid maintenance and copy number. Bacteria containing pAB120 variants with or without *higBA2*_Ab_ or both *higBA2*_Ab_ and *splTA*_Ab_ TA system were selected on media containing meropenem. Inside the box: plasmid copy number (PCN) of pAB120 variants with and without *higBA2_Ab_* was calculated after 24 h growth without antibiotic pressure. PCN was calculated as described in Section “Materials and Methods”. The experiment was independently repeated 10 times, the difference of PCN between the two strains was statistically significant (*t*-test, *p* < 0.05); **(B)** Effect of *higBA2*_Ab_ locus in stabilization of unstable plasmid pAcORI^∗^. *Acinetobacter baylyi* containing variants pAcORI^∗^ with and without *higBA2*_Ab_ were selected on gentamicin (pAcORI^∗^ marker).

Toxin-antitoxin modules are known plasmid stabilization factors in bacteria (Engelberg-Kulka and Glaser, 1999; Hernández-Arriaga et al., 2015) and their presence on plasmids conferring resistance to clinically important antibiotics may contribute to the persistence and spread of antibiotic resistant strains. We have previously shown that *higBA2*_Ab_ module is widely spread among *A. baumannii* plasmids (Povilonis et al., 2013; Sužiedėlienė et al., 2016), therefore we were interested whether it provides stabilization function for cognate plasmid carrying resistance genes, found in clinical *A. baumannii* isolates. For this purpose plasmid pAB120, which originally carries two copies of *bla_OXA-72_* gene conferring resistance to carbapenems (meropenem and imipenem) was purified from clinical *A. baumannii* strain K60 and *higBA2*_Ab_ locus was deleted (Supplementary Figure S1). Given that pAB120 plasmid is equipped with another type II TA module, *splTA* (Jurenaite et al., 2013; Povilonis et al., 2013), we sought if the latter could be sufficient to supply plasmid stabilization even when *higBA2*_Ab_ was deleted. Therefore, plasmid with deletion of both TA modules was also constructed (Supplementary Figure S1). The pAB120Δ*higBA*_Ab_ and pAB120Δ*higBA*_Ab_Δ*splTA*_Ab_ plasmids were transformed into non-pathogenic *Acinetobacter baylyi* strain ADP1, which is known not to contain any plasmids or *higBA* homologs (Supplementary Table S1). Unexpectedly, neither the deletion of *higBA2*_Ab_ locus, nor the elimination of both *higBA2*_Ab_ and *splTA*_Ab_ modules caused any loss of pAB120 variants in *A. baylyi* (not shown). To eliminate the possibility of different plasmid maintenance effects in different *Acinetobacter* species, *A. baumannii* clinical strain K53 was then used as a host (the strain also does not contain any plasmids of known *A. baumannii* replication groups and *higBA* modules (Jurenaite et al., 2013; Povilonis et al., 2013; Supplementary Table S1). pAB120 variants did not show decrease in plasmid retention in *A. baumannii* as well (**Figure 6A**). We then analyzed whether *higBA2*_Ab_ locus has an impact on the copy number of pAB120 plasmid. *A. baumannii* strains K53 bearing pAB120 with or without *higBA2*_Ab_ were grown without antibiotic pressure for 24 h and PCN was calculated by qPCR. A slight reduction in PCN was observed, which was statistically significant (**Figure 6A**). These results indicate that while *higBA2*_Ab_ and *splTA*_Ab_ are not the main players in stabilization of pAB120 under tested conditions, the deletion of *higBA2*_Ab_ has an impact on its copy number, and could influence plasmid retention in the long run or in different conditions.

To be sure if *higBA2*_Ab_ is able to confer stability when introduced into unstable plasmid lacking any stabilizing determinants, we used *higBA2*_Ab_ from pAB120 in additional plasmid stabilization experiments. The *higBA2*_Ab_ locus with its own promoter region was cloned into unstable *Acinetobacter* plasmid pAcORI^∗^, conferring resistance to gentamicin (Supplementary Table S1). *A. baylyi* ADP1 strain containing plasmid pAcORI^∗^ with *higBA2*_Ab_ locus and plasmid without the TA system were grown without antibiotic pressure and plasmid retention was calculated. Strikingly, in this background the presence of *higBA2*_Ab_ locus on the pAcORI^∗^ plasmid ensured its stability when bacteria were grown for over 80 h (**Figure 6B**). This result together with the previous observations of the effect on PCN confirms that *higBA2*_Ab_ module can play a role in plasmid maintenance.

## Discussion

The TA modules which are grouped into *higBA* family (*host inhibition of growth*) encode a RelE-like toxin and antitoxin that contains a HTH Xre-domain (Gerdes et al., 2005; Makarova et al., 2009). Toxins of HigB family belong to a large RelE/ParE superfamily consisting of mRNAses such as RelE toxin as well as gyrase poisons such as ParE toxin (Anantharaman and Aravind, 2003). Within this superfamily, GP49-domain toxins show low but significant homology to RelE (Makarova et al., 2009). TA modules containing GP49-domain toxin in all studied cases constitute the reverse type of TA operons where toxin is encoded first and is followed by an antitoxin (Dziewit et al., 2007; Ramage et al., 2009; Jurenaite et al., 2013; Sala et al., 2014). Despite being classified as HigB family toxins, the GP49-domain toxins show little similarity to well-studied HigB toxins from *E. coli, Vibrio cholerae, Proteus vulgaris* (Tian et al., 1996; Christensen-Dalsgaard and Gerdes, 2006; Christensen-Dalsgaard et al., 2010) as well as to toxins of other TA families of RelE superfamily. HigBA_Ab_ shows only ∼20% sequence identity to other validated TA modules that have GP49-domain toxins and were named as Tad toxins (Dziewit et al., 2007).

pAB120 plasmid-borne HigBA2_Ab_ proteins form strong complex with molecular mass of approximately 100 kDa suggesting that up to eight protein molecules might be present within the oligomer (the predicted molecular masses of HigB2_Ab_ and HigA2_Ab_ proteins are 13.5 and 11.2 kDa, respectively). According to the currently available structural data, the RelE toxin-based TA superfamily TA complexes are known to form heterotrimers (*E. coli* YefM-YeoB, PDB accession: 2A6Q), and most commonly, heterotetramers (*E. coli* DinJ-YafQ, PDB accession: 4Q2U; MqsR-MqsA, PDB accession: 3HI2, *E. coli* RelB-RelE, PDB accession: 4FXE; *Brucella abortus* BrnT-BrnA (Heaton et al., 2012), *Proteus vulgaris* HigBA (Schureck et al., 2014)]. In some cases complexes crystallize as more complex oligomeric structures: a so far unique hetero-hexadecameric complex has been observed for ParE2-PaaA2 TA system (Sterckx et al., 2016). ParE-family toxins inhibit DNA replication and despite sharing common fold they are functionally different from RelE-family toxins. Therefore *A. baumannii* HigBA2_Ab_ complex of 100 kDa, the size strongly exceeding that of a heterotetramer, observed in this study might represent one of the most complex oligomeric structures known for RelE family TAs.

Taking into account the organization of *A. baumannii higBA2*_Ab_ module, where toxin gene is located upstream the antitoxin gene, a feature known for a limited number of TAs (Christensen-Dalsgaard and Gerdes, 2006), the expression of equal amounts of both proteins must be ensured in the cell to avoid a harmful effect of the toxin. According to our observations, equal levels of *higB2*_Ab_ and *higA2*_Ab_ transcripts are produced at the standard growth conditions in *A. baumannii* indicating that TA balance at least at the transcription level is properly preserved. The transcript of the whole operon (spanning both *higB2*_Ab_ and *higA2*_Ab_ genes) could also be detected by qPCR (Armalytė, Unpublished data), whereas an additional promoter for the *higA2*_Ab_ gene was not, indicating that most likely *higBA2*_Ab_ is transcribed as a single transcript. HigA2_Ab_ antitoxin and HigBA2_Ab_ complex autorepressed their own promoter, indicating the transcriptional regulatory mode common to type II TA operons in other bacteria (Chan et al., 2016). HigA2_Ab_ antitoxin harbors HTH Xre-like domain, which is present in some antitoxins of type II TAs such as HigA from *Proteus vulgaris*, and has been shown to bind operator sequences which overlap with the promoter region (Schureck et al., 2014). The more pronounced inhibition by HigBA2_Ab_ complex than by single antitoxin indicates HigB2_Ab_ toxin also takes part in transcriptional autoregulation of the system.

Despite well studied transcriptional regulation for TA systems, the data on their expression *in vivo* in conditions relevant to bacterial lifestyle, in particular to that of pathogenic species, is only beginning to emerge. *A baumannii* represents interesting example to study as it is well adapted to colonize the abiotic (glass, plastic, and metal) and biotic (human skin and mucous membranes) surfaces, withstand prolonged periods of dryness, treatment by disinfectants, antibiotics and nutrient restriction (Giannouli et al., 2013). In the host, *A. baumannii* encounters responses mediated by complement and professional and non-professional phagocytic cells. Among stress factors playing essential role in the host defense is the nutritional immunity exerted by the iron limitation (McConnell et al., 2013). Our data suggest that stationary growth and iron depletion causes the induction of both toxin and antitoxin genes of *higBA2*_Ab_ module at a similar level, therefore, expression of both components could play a role for *A. baumannii* stress response. This is supported by recent observation that closest homologues of *A. baumannii higBA* in *M. tuberculosis*, Rv2022c-Rv2021c (*higBA2*) and Rv3182-Rv3183 (*higBA3*) are among the strongest stress responsive modules of this pathogen, activated in response to multiple stressors including antibiotics, starvation and low pH (Gupta et al., 2017). In our study, we found that only rifampicin influenced the expression of *higBA2*_Ab_ by reducing it, which was the opposite effect from that observed for *M. tuberculosis higBA*s. Sub-inhibitory concentrations of antibiotics are known to induce variable gene expression outcomes in bacteria. SOS response is commonly induced, which includes upregulation of stress proteases such as Lon and Clp. Generally they target antitoxins and relieve transcriptional repression of TAs (Muthuramalingam et al., 2016). However, the observations that particular TAs are downregulated after antibiotic exposure indicate that specific inhibition of the activities of the TA promoters could be involved. Type II loci of other human pathogens have been recently shown to transcriptionally respond under stress conditions. *H. pylori hp0893-hp0892* module, which is the most prevalent TA system among *H. pylori* clinical isolates was induced during stationary growth, in the low concentration of iron and nickel as well as in a high concentration of urea, conditions mimicking the host environment, suggesting that this TA gene pair might represent a novel *H. pylori* stress responsive virulence factor (Cárdenas-Mondragón et al., 2016). Intracellular pathogen *B. abortus* increased the expression of type II *brnTA* system in the presence of chloramphenicol, H_2_O_2_ and low pH stress (Heaton et al., 2012). In *S. enterica* type II s*ehAB* module responded to minimal medium and within macrophages (De la Cruz et al., 2013), whereas different expression of some toxins of type II systems has been observed inside fibroblasts and epithelial cells (Lobato-Márquez et al., 2015).

The induction of bacterial TAs in stress conditions has been attributed to the enhanced degradation of antitoxin by stress peptidases or decreased translation under stress conditions (Ramisetty et al., 2016). This, in turn, might result in the derepression of transcription of TA operon to a different level depending on the stress influence on antitoxin proteolysis and translation rates. Therefore, the decrease in antitoxin concentration might result in various different physiological outcomes: the toxin, freed from the complex, could quickly come into action leading to growth reduction, or an increased transcriptional response of TA genes could be induced, led by derepression of the operon. Such a range in TA-mediated outcomes would be beneficial to *A. baumannii* in contributing to its persistence in clinical environment and in sensing the host.

The *higBA*_Ab_ locus, with a few exceptions, is found so far on the *A. baumannii* plasmids, which frequently harbor antibiotic resistance genes coding for carbapenemases OXA-72, OXA-23, and OXA-58 (Sužiedėlienė et al., 2016). We found that 67 out of 307 (21.8%) *Acinetobacter* sp. plasmids sequenced to date (GenBank accessed 2018/01/11) had *higBA*_Ab_ TA systems. The location of *higBA*_Ab_ could not be attributed to a certain plasmid replication group or plasmid size (plasmids sizes varied from 7.4 to 121 kb). The high prevalence of these TA systems indicates their easy spread and stable maintenance on the plasmids. The mechanisms responsible for plasmid stabilization in *A. baumannii* are largely unknown (Lean and Yeo, 2017) and this study is the first attempt to assess the role of TA systems in *A. baumannii*. Surprisingly, we have shown that despite the ability of *higBA2*_Ab_ module to function as a *bone fide* addiction system by supporting maintenance of unstable plasmid in *Acinetobacter*, it did not play that role for pAB120 plasmid from clinical *A. baumannii* strain, as did not another type II module, carried by the same plasmid. This data suggest that other elements than plasmid-borne post-segregational killing pathway components are responsible for pAB120 stabilization. Among the possible candidates are XerC/XerD sites, which are known to participate in recombinase-based proper resolution of plasmid copies (Sengupta and Austin, 2011) and are present in pAB120 plasmid in multiple copies as well as in series of *A. baumannii* plasmids of the same replicon type (Lean and Yeo, 2017).

Another unexpected observation was the detection of several *higBA*_Ab_ copies in plasmids and *A. baumannii* isolates in the sequence databases. Interestingly, only the less conserved *higBA2*_Ab_ group was detected in that setting, and we were unable to find both groups *higBA*_Ab_ in one isolate or plasmid in the pool of *Acinetobacter* sp. sequences, available to this day. In agreement with this observation we have demonstrated that both groups’ antitoxins are able to counteract the toxins. Such counter-activity has been reported for other TA systems (Zhu et al., 2010). In case of HigBA_Ab_, the two versions might work as counter-addiction modules for each other. The observed duplication of *higBA2*_Ab_ could indicate the ongoing evolution and divergence of the novel TA species.

## Author Contributions

JA, DJ, RK, and ES designed the experiments. JA, DJ, RK, and AČ performed the experiments. JA, DJ, and ES analyzed the data and wrote the paper.

## Conflict of Interest Statement

The authors declare that the research was conducted in the absence of any commercial or financial relationships that could be construed as a potential conflict of interest.
