# Supplementary material for: The higBA Toxin-Antitoxin Module From the Opportunistic Pathogen Acinetobacter baumannii – Regulation, Activity, and Evolution
Source: Front Microbiol. 2018 Apr 12;9:732. doi: 10.3389/fmicb.2018.00732 (PMC5906591; doi:10.3389/fmicb.2018.00732)
Supplement: Supplementary file 1 [file Table_1.DOCX]

Supplementary Material

The *higBA* Toxin-Antitoxin Module from the Opportunistic Pathogen *Acinetobacter baumannii* – Regulation, Activity and Evolution

Julija Armalytė*, Dukas Jurėnas, Renatas Krasauskas, Albinas Čepauskas, Edita Sužiedėlienė

*** Correspondence:** Julija Armalytė: julija.armalyte@gf.vu.lt

**Table S1.** Plasmid and bacterial strains used in the study.

| Bacterial strain/plasmid | Description | Reference |
| --- | --- | --- |
| **Strains** |  |  |
| *Escherichia* *coli* |  |  |
| BW25113 F’ | *lacI^+^rrnB_T14_ ΔlacZ_WJ16_hsdR514 ΔaraBAD_AH33_ ΔrhaBAD_LD78_rph-1 Δ(araB–D)567 Δ(rhaD–B)568 ΔlacZ4787(::rrnB-3) hsdR514rph-1/F’proA^+^B^+^lacIqΔlacZ)M15 zzf::mini-Tn10 (Kan^r^)* | (Motiejūnaite et al., 2007) |
| BTH101 | *cya-99, araD139, galE15, galK16, rpsL1 (Str^r^), hsdR2, mcrA1, mcrB1* | Euromedex |
| BL21(DE3) | *ompTgaldcmlonhsdS_B_*(*r_B_*^–^*m_B_*^–^) λ(DE3 [*lacI lacUV5*-*T7p07ind1sam7nin5*]) [*malB*^+^]_K-12_(λ^S^) | (Studier and Moffatt, 1986) |
| DJ624*∆ara* | MG1655 *lacX74 mal::lacI^q^* | (Hallez et al., 2010) |
| *Acinetobacter baumannii* |  |  |
| K60 | Clinical isolate, ECII | (Povilonis et al., 2013) |
| K53 | Clinical isolate | (Povilonis et al., 2013) |
| 35 | Clinical isolate, ECII | (Povilonis et al., 2013) |
| *Acinetobacter baylyi* ADP1 |  | ATCC 33305 |
|  |  |  |
| **Plasmids** |  |  |
| pAB120 | 11 kb *A. baumannii* plasmid from clinical isolate | (Povilonis et al., 2013) |
| pAB120*∆*higBA | *higBA2* TA system deleted from pAB120 | This work |
| pPROBE-gfp (tagless) | Plasmid containing promoterless *gfp* gene | (Miller et al., 2000) |
| pPROBE-PhigBA-gfp | 200 bp of *higBA2*_Ab_ promoter region cloned to pPROBE-gfp(tagless) | This work |
| pPROBE-PhigA-gfp | 200 bp of predicted *higA2*_Ab_ promoter region cloned to pPROBE-gfp(tagless) | This work |
| pBAD24 | Expression plasmid | (Guzman et al., 1995) |
| pBAD24-HigBA | *higBA2*_Ab_ cloned to pBAD24 | This work |
| pBAD24-HigA | *higA2*_Ab_ cloned to pBAD24 | This work |
| pBAD30 | Expression plasmid | (Guzman et al., 1995) |
| pBAD-GP49_Ab_ | *higB1_Ab_* cloned to pBAD30 | (Jurenaite et al., 2013) |
| pBAD-higBpAB120 | *higB2_Ab_* cloned to pBAD30 | This work |
| pUHEcat | Expression plasmid | (Motiejūnaite et al., 2007) |
| pUHEcat-Cro_Ab_ | *higA1_Ab_* cloned to pUHEcat | (Jurenaite et al., 2013) |
| pUHEcat-higApAB120 | *higA2_Ab_* cloned to pUHEcat | This work |
| pWH1266 | *Acinetobacter calcoaceticus* plasmid fragment containing ORI cloned to pBR322 | (Hunger et al., 1990) |
| pAcORI* | pWH1266 plasmid fragment cloned to pUC19 with introduced gm^R^ | This work |
| pAcORI*higBA | *higBA2*_Ab_ with own promoter cloned to pAcORI* | This work |
| pUT18 | Plasmid for BACTH assay N-terminal fusions with *cya*T18 | Euromedex |
| pKNT25 | Plasmid for BACTH assay N-terminal fusions with *cya*T25 | Euromedex |
| pUT18-RelB | *A. baumannii relB* N-terminal fusion to *cya* T18 | This work |
| pKNT25-RelE | *A. baumannii relE* N-terminal fusion to *cya* T25 | This work |
| pUT18-HigA | *A. baumannii higA2* N-terminal fusion to *cya* T18 | This work |
| pKNT25-HigB | *A. baumannii higB2* N-terminal fusion to *cya* T25 | This work |
| pET28b | Protein expression vector | Novagen |
| pET-HigBA-His | HigBA2 expression plasmid, His-tag fused C-terminally to antitoxin | This work |
| pET-His-HigBA | HigBA2 expression plasmid, His-tag fused N-terminally to toxin | This work |
| pET-His6-TEV-HigB-HigA | HigBA2 expression plasmid, His-tag and TEV site fused N-terminally to toxin | This work |
| pET-HigB-His6-TEV-HigA | HigBA2 expression plasmid, His-tag and TEV site fused N-terminally to antitoxin | This work |

## References

Guzman, L. M., Belin, D., Carson, M. J., and Beckwith, J. (1995). Tight regulation, modulation, and high-level expression by vectors containing the arabinose PBAD promoter. *J. Bacteriol.* 177, 4121–4130.

Hallez, R., Geeraerts, D., Sterckx, Y., Mine, N., Loris, R., and Van Melderen, L. (2010). New toxins homologous to ParE belonging to three-component toxin–antitoxin systems in Escherichia coli O157:H7. *Mol. Microbiol.* 76, 719–732. doi:10.1111/j.1365-2958.2010.07129.x.

Hunger, M., Schmucker, R., Kishan, V., and Hillen, W. (1990). Analysis and nucleotide sequence of an origin of DNA replication in Acinetobacter calcoaceticus and its use for Escherichia coli shuttle plasmids. *Gene* 87, 45–51.

Jurenaite, M., Markuckas, A., and Suziedeliene, E. (2013). Identification and characterization of type II toxin-antitoxin systems in the opportunistic pathogen Acinetobacter baumannii. *J. Bacteriol.* 195, 3165–3172. doi:10.1128/JB.00237-13.

Miller, W. G., Leveau, J. H., and Lindow, S. E. (2000). Improved gfp and inaZ broad-host-range promoter-probe vectors. *Mol. Plant-Microbe Interact. MPMI* 13, 1243–1250. doi:10.1094/MPMI.2000.13.11.1243.

Motiejūnaite, R., Armalyte, J., Markuckas, A., and Suziedeliene, E. (2007). Escherichia coli dinJ-yafQ genes act as a toxin-antitoxin module. *FEMS Microbiol. Lett.* 268, 112–119.

Povilonis, J., Seputiene, V., Krasauskas, R., Juskaite, R., Miskinyte, M., Suziedelis, K., et al. (2013). Spread of carbapenem-resistant Acinetobacter baumannii carrying a plasmid with two genes encoding OXA-72 carbapenemase in Lithuanian hospitals. *J. Antimicrob. Chemother.* 68, 1000–1006. doi:10.1093/jac/dks499.

Studier, F. W., and Moffatt, B. A. (1986). Use of bacteriophage T7 RNA polymerase to direct selective high-level expression of cloned genes. *J. Mol. Biol.* 189, 113–130.
