# Supplementary material for: The higBA Toxin-Antitoxin Module From the Opportunistic Pathogen Acinetobacter baumannii – Regulation, Activity, and Evolution
Source: Front Microbiol. 2018 Apr 12;9:732. doi: 10.3389/fmicb.2018.00732 (PMC5906591; doi:10.3389/fmicb.2018.00732)
Supplement: Supplementary file 2 [file Table_2.DOCX]

Supplementary Material

The *higBA* Toxin-Antitoxin Module from the Opportunistic Pathogen *Acinetobacter baumannii* – Regulation, Activity and Evolution

Julija Armalytė*, Dukas Jurėnas, Renatas Krasauskas, Albinas Čepauskas, Edita Sužiedėlienė

*** Correspondence:** Julija Armalytė: julija.armalyte@gf.vu.lt

**Table S2**. Primers used in the study

| Primer | Sequence 5’-3’ | Description |
| --- | --- | --- |
| F_HigB | Gatgtggacagtcattacaacg | Cloning of *higB2_Ab_* to pBAD30 |
| R_HigB | atgcAAGCTTgttgaatacgagcctgac | Cloning of *higB2_Ab_* to pBAD30, HindIII site underlined |
| F_HigA | Gatggctaagaccctacaaga | Cloning of *higA2_Ab_* to pUHEcat |
| R_HigA | gactAAGCTTttctaggctatcggcagca | Cloning of *higA2_Ab_* to pUHEcat, HindIII site underlined |
| higBA_F | agccatgggttaaacagatccgttgtaatg | Deletion of *higBA2* from pAB120, introduced NcoI sites underlined |
| higBA_R | atccatggagagcttccaacaggaaaac | Deletion of *higBA2* from pAB120, introduced NcoI sites underlined |
| Ac_ORI_F | gatcaagcttgatcgtagaaatatctatgattat | Cloning of partial *Acinetobacter* sp. ORI from pWH1266, HindIII underlined |
| R_Ac-ori1273 | atttatctgcaggtatacattcatttt | Cloning of partial *Acinetobacter* sp. ORI from pWH1266, PstI underlined |
| F_opHigBA | ttgcggatccatcttgggataaatat | Cloning of *higBA2* with own promoter to pAcORI*, BamHI site underlined |
| R_opHigBA | atatgagctcgtccaagttaacaagt | Cloning of *higBA2* with own promoter to pAcORI*, SacI underlined |
| RelT_pUpKN_F | CCActgcagcATGAGTTACGAGCTAGAGTT | Cloning *A. baumannii relT* to pUT18, PstI underlined |
| RelT_pUpKN_R | GAggatcctcCATCTGTTTATAGATAACATCTC | Cloning *A. baumannii relT* to pUT18, BamHI undelined |
| RelA_pUpKN_F | CCActgcagcATGAGAAACGTCATGAACCAC | Cloning *A. baumannii relA* to pKNT25, PstI underlined |
| RelA_pUpKN_R | GaggatccgaAGAGCTGTTTTTGAGAACTCT | Cloning *A. baumannii relA* to pKNT25, BamHI underlined |
| HigT_F | CCACTGCAGAATGTGGACAGTCATTACAACG | Cloning of *higB2* to pKNT25, PstI underlined |
| HigT_R | GAGGATCCACGCCATTAGATTGATCTCCTAA | Cloning *higB2* to pKNT25, BamHI undelined |
| HigA_F | CCACTGCAGAATGGCTAAGACCCTACAAGA | Cloning *higA2* to pUT18, PstI underlined |
| HigA_R | GAGGATCCACGACATTAAATCCTATGTGTTTTC | Cloning *higA2* to pUT18, BamHI underlined |
| higB_FpET | gatcCCATGGggacagtcattacaacggat | Cloning of *higBA2* to pET28b, fusing his-tag to antitoxin, NcoI underlined |
| higA_RpET | gatcCTCGAGgacattaaatcctatgtgttttc | Cloning of *higBA2* to pET28b, fusing his-tag to antitoxin, XhoI underlined |
| pET28_higBA­_F_N | acCATATGtggacagtcattacaacggatct | Cloning of *higBA2* to pET28b, fusing his-tag to toxin, NdeI underlined |
| pET28_higBA_R_N | tcCTCGAGttagacattaaatcctatgtgttt | Cloning of *higBA2* to pET28b, fusing his-tag to toxin, XhoI underlined |
| pET_TEV_higB_F | ATGTGGACAGTCATTACAACG | Introduction of TEV site into pET-His-HigBA by inverse PCR |
| pET_TEV_higB_R | AGACTGGAAGTACAGGTTTTCGCCGCTGCTGTGATGATG | Introduction of TEV site into pET-His-HigBA by inverse PCR |
| pET_remTEV_higB_F | CCATGTGGACAGTCATTACAAC | Removal of TEV site from pET-His6-TEV-HigB-HigA by inverse PCR |
| pET_remTEV_higB_R | TATATCTCCTTCTTAAAGTTAAACAAAAT | Removal of TEV site from pET-His6-TEV-HigB-HigA by inverse PCR |
| pET_TEV_higA_F | GCCGCTGCTGTGATGATGATGATGATGCTTAGCCATTAGATTGATCTCCTA | Introduction of TEV site in the N-terminal part of *higA2* by inverse PCR |
| pET_TEV_higA_R | GAAAACCTGTACTTCCAGTCTGCTAAGACCCTACAAGAATTATTGGC | Introduction of TEV site in the N-terminal part of *higA2* by inverse PCR |
| F-PhigBA-SacI | GACTGAGCTCGACACATAGAGAGCAGGAT | Cloning *higBA2* promoter to pPROBE-gfp, SacI underlined |
| R-PhigBA-BamHI | GATCGGATCCATTACAACCATAATATAGATTACAGT | Cloning *higBA2* promoter top pPROBE-gfp, BamHI underlined |
| F-PhigA-SacI | GATCGAGCTCCAAATTTACCAATATGAAAGA | Cloning putative *higA2* promoter to pPROBE-gfp, SacI underlined |
| R-PhigA-BamHI | GATCGGATCCTAGATTGATCTCCTAAAGTTGAG | Cloning putative *higA2* promoter to pPROBE-gfp, BamHI underlined |
| F-HigB-EcoRI | GATCGAATTCATGTGGACAGTCATTACAAC | Cloning *higBA2* to pBAD24, EcoRI underlined |
| F-HigA-EcoRI | GATCGAATTCATGGCTAAGACCCTACAAG | Cloning *higA2* to pBAD24, EcoRI underlined |
| R-HigA-HindIII | GATCAAGCTTTTAGACATTAAATCCTATGTGTTT | Cloning *higBA2* or *higA2* to pABD24, HindIII underlined |
| Rep_qF | cgttgggtatctcaaattgc | qPCR primers for plasmid copy number detection |
| Rep_qR | gaatggcataagcgctacta | qPCR primers for plasmid copy number detection |
| rpoB_qF | cgattcgtacagaacattctt | qPCR house-keeping gene primers |
| rpoB_qR | taaagcagcattgccagaata | qPCR house-keeping gene primers |
| Hig_qF | cgtcctttagtagataccgt | qPCR *higB* gene expression |
| Hig_qR | cagcacaatagcttgtcgta | qPCR *higB* gene expression |
| HigA_qF | tgttggaaactcagcttcac | qPCR *higA* gene expression |
| HigA_qR | gatcatgaccacgattttcg | qPCR *higA* gene expression |
| Tuf_qF | gaagcgaaagattactcacaa | qPCR *tuf* gene expression |
| Tuf_qR | cagtgatcatgtttttaacgta | qPCR *tuf* gene expression |
