# Supplementary material for: The higBA Toxin-Antitoxin Module From the Opportunistic Pathogen Acinetobacter baumannii – Regulation, Activity, and Evolution
Source: Front Microbiol. 2018 Apr 12;9:732. doi: 10.3389/fmicb.2018.00732 (PMC5906591; doi:10.3389/fmicb.2018.00732)
Supplement: Supplementary file 3 [file Table_3.DOCX]

Supplementary Material

The *higBA* Toxin-Antitoxin Module from the Opportunistic Pathogen *Acinetobacter baumannii* – Regulation, Activity and Evolution

Julija Armalytė*, Dukas Jurėnas, Renatas Krasauskas, Albinas Čepauskas, Edita Sužiedėlienė

*** Correspondence:** Julija Armalytė: julija.armalyte@gf.vu.lt

**Table S3.** *Acinetobacter* sp. plasmids, containing two *higBA*_Ab_ versions. *higBA*_Ab_ genes are indicated in blue and cyan, respectively. The maps were prepared by SnapGene.

| \| Accession number \| Size, kb \| Plasmid name and comments \| Plasmid map \| \| --- \| --- \| --- \| --- \| \| CM008888 \| 8.24 \| *Acinetobacter baumannii* strain ZQ1 plasmid unnamed2  Two identical *higBA2*_Ab_ \|  \| \| CP010369 \| 89.11 \| *Acinetobacter nosocomialis* strain 6411 plasmid p6411  Two different *higBA2*_Ab_ versions \| 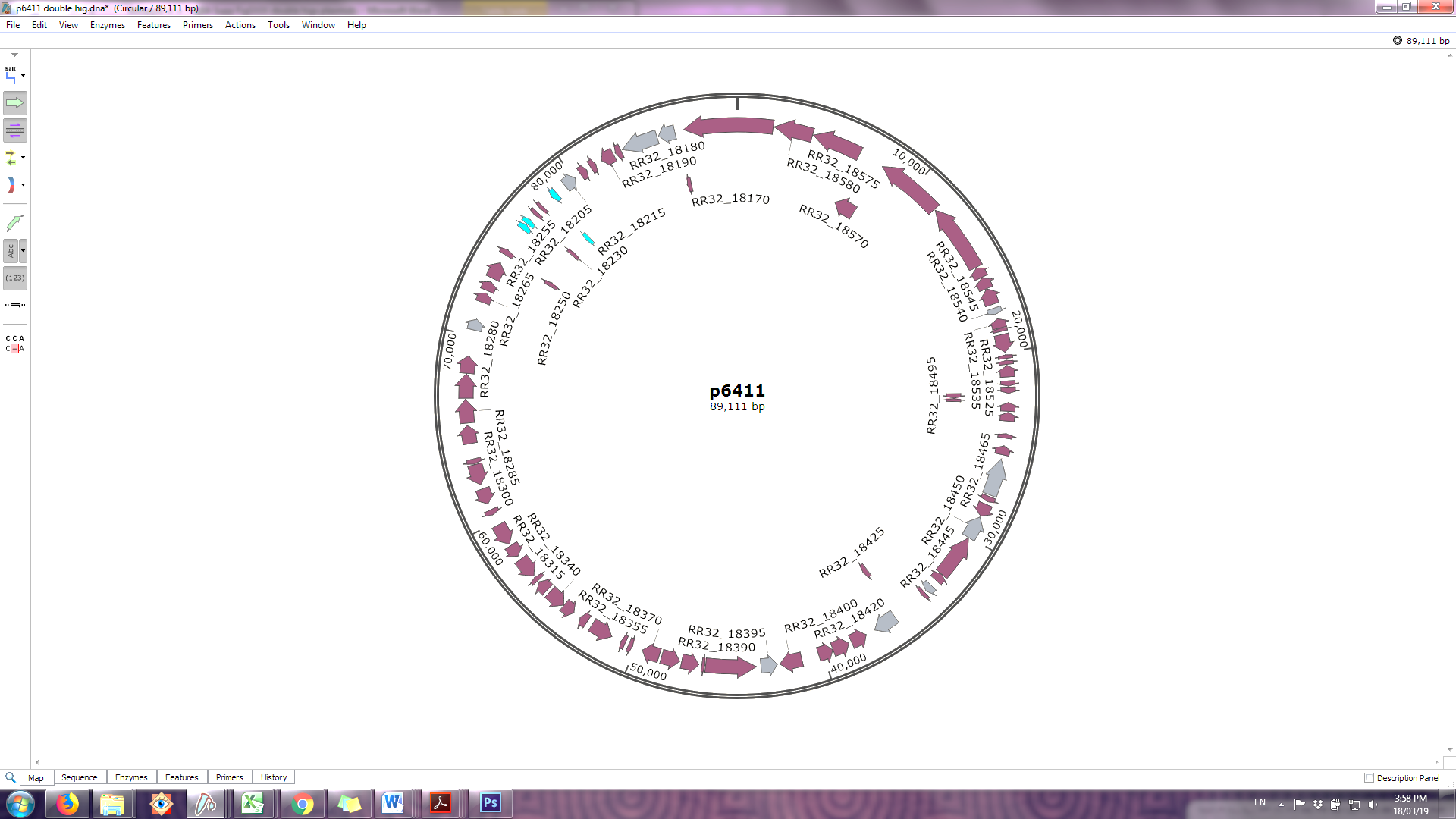 \| \|  \|  \|  \|  \| \| CM001803 \| 93.89 \| *Acinetobacter calcoaceticus* subsp. *anitratus* XM1570 plasmid pXM2  Two different *higBA2*_Ab_ versions \|  \| \| NC_010404 \| 94.41 \| *Acinetobacter baumannii* str. AYE plasmid p3ABAYE  Two different *higBA2*_Ab_ versions \|  \| \|  \|  \|  \|  \| \| NC_025173 \| 94.42 \| *Acinetobacter pittii* strain MS32 plasmid pMS32-1  Two different *higBA2*_Ab_ versions \|  \| |
| --- | --- | --- | --- | --- | --- | --- | --- | --- | --- | --- | --- | --- | --- | --- | --- | --- | --- | --- | --- | --- | --- | --- | --- | --- | --- | --- | --- | --- | --- | --- | --- | --- |
|  |
