# Supplementary material for: The higBA Toxin-Antitoxin Module From the Opportunistic Pathogen Acinetobacter baumannii – Regulation, Activity, and Evolution
Source: Front Microbiol. 2018 Apr 12;9:732. doi: 10.3389/fmicb.2018.00732 (PMC5906591; doi:10.3389/fmicb.2018.00732)
Supplement: Supplementary file 4 [file Table_4.DOCX]

Supplementary Material

The *higBA* Toxin-Antitoxin Module from the Opportunistic Pathogen *Acinetobacter baumannii* – Regulation, Activity and Evolution

Julija Armalytė*, Dukas Jurėnas, Renatas Krasauskas, Albinas Čepauskas, Edita Sužiedėlienė

*** Correspondence:** Julija Armalytė: julija.armalyte@gf.vu.lt

**Table S4.** The MIC values of *A. baumannii* K60 strain.

| Antibiotic | MIC, μg/mL |
| --- | --- |
| Rifampicin | 3.125 |
| Streptomycin | >1000 |
| Gentamicin | 20 |
| Kanamycin | 640 |
| Meropenem | 32 |
| Cefuroxime | 256 |
| Ciprofloxacin | >16 |
| Tetracyclin | >200 |
