# Supplementary material for: The higBA Toxin-Antitoxin Module From the Opportunistic Pathogen Acinetobacter baumannii – Regulation, Activity, and Evolution
Source: Front Microbiol. 2018 Apr 12;9:732. doi: 10.3389/fmicb.2018.00732 (PMC5906591; doi:10.3389/fmicb.2018.00732)
Supplement: Supplementary file 5 [file Data_Sheet_1.DOCX]

Supplementary Material

The *higBA* Toxin-Antitoxin Module from the Opportunistic Pathogen *Acinetobacter baumannii* – Regulation, Activity and Evolution

Julija Armalytė*, Dukas Jurėnas, Renatas Krasauskas, Albinas Čepauskas, Edita Sužiedėlienė

*** Correspondence:** Julija Armalytė: julija.armalyte@gf.vu.lt

|  |
| --- |
| **Figure S1.** pAB120 plasmid map. XerC/XerD-like sites are indicated as Xer, the deletion regions of *higBA2*_Ab_ and *splTA*_Ab_ are indicated as grey boxes. *higBA2*_Ab_ was deleted by inverse PCR, as indicated in the Methods section. As we were unable to use the same approach on *splTA*_Ab_ (the vicinity of the operon contains repetitive sequences therefore it is impossible to create unique primers), the TA system together with *tonB* gene (indicated as pAB120_08 in the map) was deleted by restriction of pAB120 with Eco32I (Thermo Fisher Scientific), and re-ligation of the plasmid part without *splTA*_Ab_. The map was generated using SnapGene |
