# Supplementary material for: The higBA Toxin-Antitoxin Module From the Opportunistic Pathogen Acinetobacter baumannii – Regulation, Activity, and Evolution
Source: Front Microbiol. 2018 Apr 12;9:732. doi: 10.3389/fmicb.2018.00732 (PMC5906591; doi:10.3389/fmicb.2018.00732)
Supplement: Supplementary file 6 [file Data_Sheet_2.DOCX]

Supplementary Material

The *higBA* Toxin-Antitoxin Module from the Opportunistic Pathogen *Acinetobacter baumannii* – Regulation, Activity and Evolution

Julija Armalytė*, Dukas Jurėnas, Renatas Krasauskas, Albinas Čepauskas, Edita Sužiedėlienė

*** Correspondence:** Julija Armalytė: julija.armalyte@gf.vu.lt

| **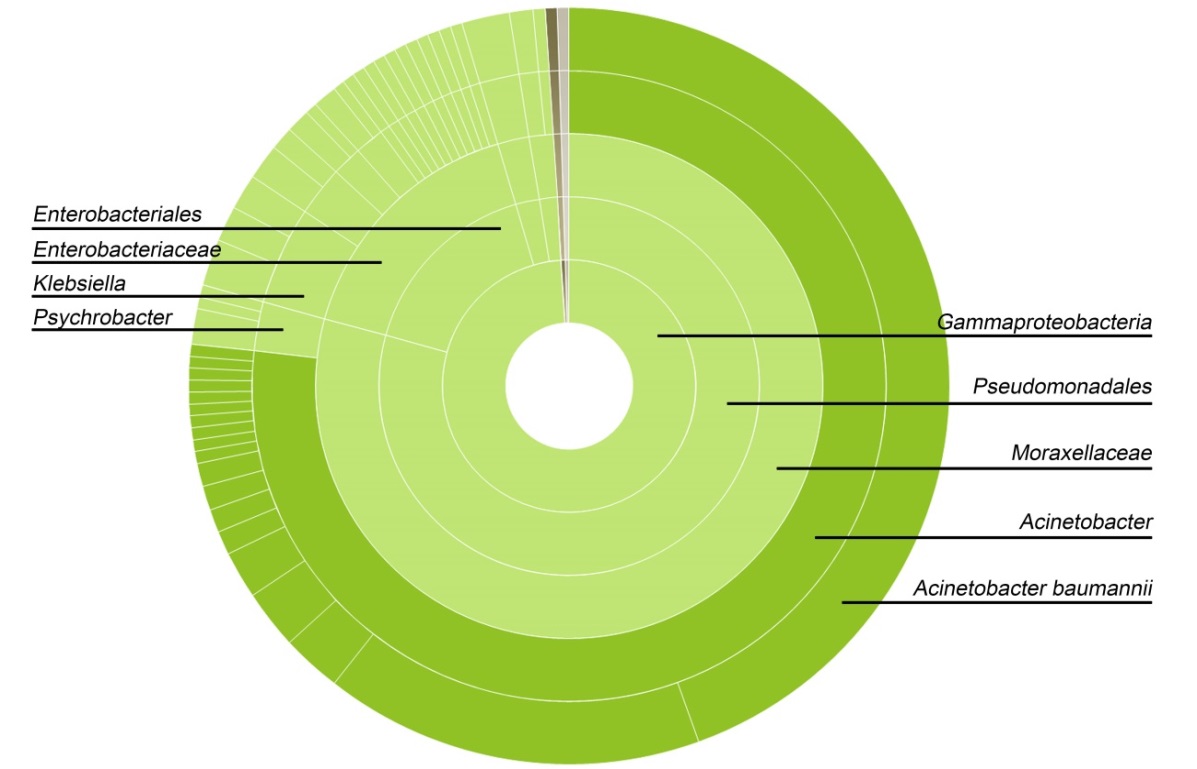** |
| --- |
| **Figure S2.** Prevalence of HigB_Ab_ toxins in bacteria. BLAST was performed in UniProt database using BLOSUM62 matrix, 200 hits represented in sunburst chart. *Acinetobacter* genus is indicated in dark green. HigB_Ab_ from pAB120 was used as a query. |
