# Supplementary material for: The higBA Toxin-Antitoxin Module From the Opportunistic Pathogen Acinetobacter baumannii – Regulation, Activity, and Evolution
Source: Front Microbiol. 2018 Apr 12;9:732. doi: 10.3389/fmicb.2018.00732 (PMC5906591; doi:10.3389/fmicb.2018.00732)
Supplement: Supplementary file 7 [file Data_Sheet_3.DOCX]

Supplementary Material

The *higBA* Toxin-Antitoxin Module from the Opportunistic Pathogen *Acinetobacter baumannii* – Regulation, Activity and Evolution

Julija Armalytė*, Dukas Jurėnas, Renatas Krasauskas, Albinas Čepauskas, Edita Sužiedėlienė

*** Correspondence:** Julija Armalytė: julija.armalyte@gf.vu.lt

| 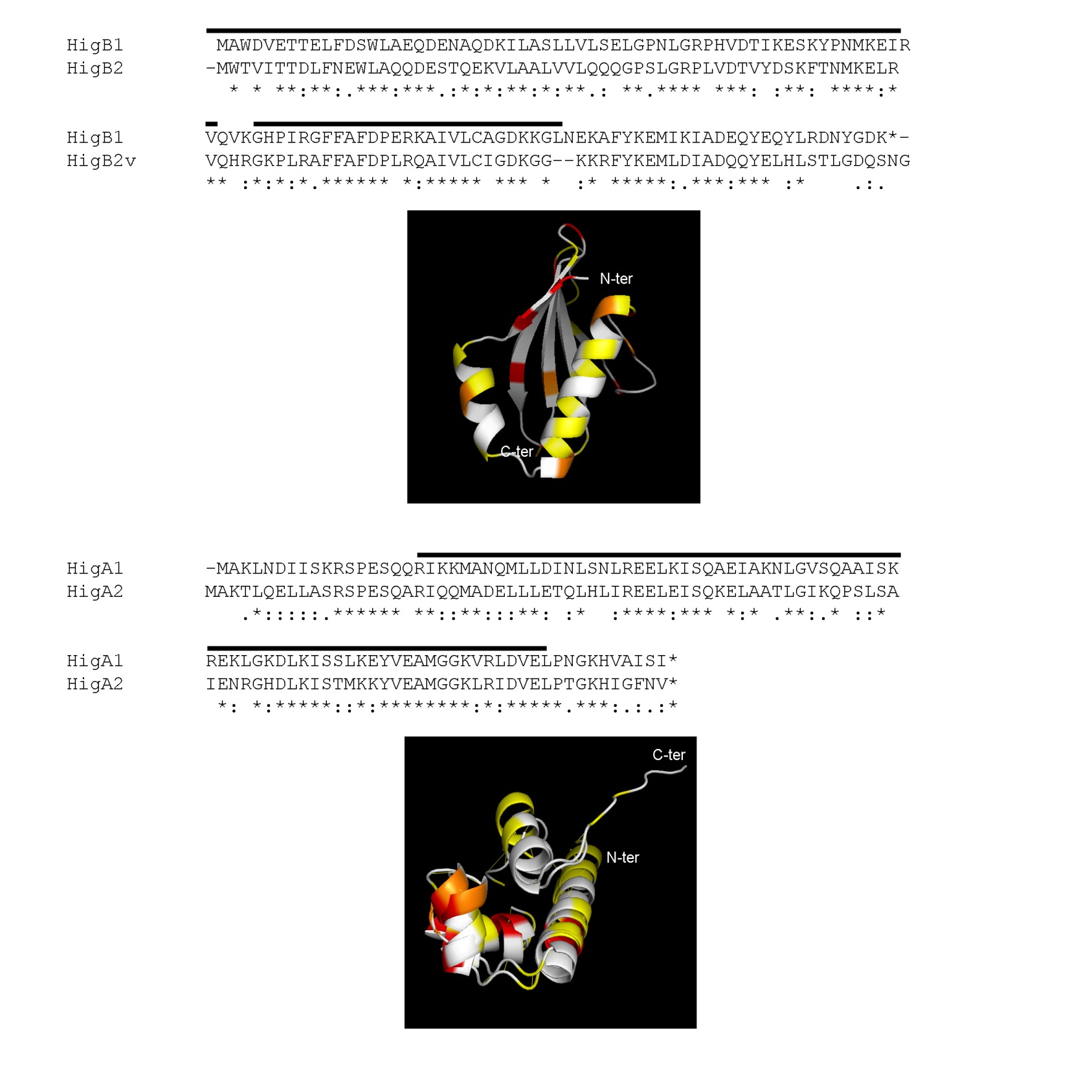 |
| --- |
| **Figure S3.** Alignment of protein structure models of the two variants of HigB_Ab_ and HigA_Ab_. Structures were modelled using PHYRE2 (Kelley et al., 2015) engine and aligned using PyMOL Viewer. Modelled regions are indicated by black lines on top of amino acid sequence alignments (Clustal Omega). Predicted protein folds are colored based on conservation – white indicates identical amino acids (* in the alignment), yellow – similar sidechains (: in the alignment), orange – partly similar sidechains (. in the alignment), red – dissimilar sidechains. HigB_Ab_s represent well conserved central beta-sheet with more differences on its extremities and the two alpha helices. The C terminus of HigB_Ab_s possibly contains additional unique extension that could not be modelled. HigA1_Ab_ and HigA2_Ab_ present most differences in the sidechains of the Cro-like DNA binding domain (N terminus), while their C terminus albeit poorly modelled and likely disordered retains good conservation. |

Reference:

Kelley, L. A., Mezulis, S., Yates, C. M., Wass, M. N., and Sternberg, M. J. E. (2015). The Phyre2 web portal for protein modeling, prediction and analysis. *Nat. Protoc.* 10, 845–858. doi:10.1038/nprot.2015.053.
