# Supplementary material for: The higBA Toxin-Antitoxin Module From the Opportunistic Pathogen Acinetobacter baumannii – Regulation, Activity, and Evolution
Source: Front Microbiol. 2018 Apr 12;9:732. doi: 10.3389/fmicb.2018.00732 (PMC5906591; doi:10.3389/fmicb.2018.00732)
Supplement: Supplementary file 8 [file Data_Sheet_4.DOCX]

Supplementary Material

The *higBA* Toxin-Antitoxin Module from the Opportunistic Pathogen *Acinetobacter baumannii* – Regulation, Activity and Evolution

Julija Armalytė*, Dukas Jurėnas, Renatas Krasauskas, Albinas Čepauskas, Edita Sužiedėlienė

*** Correspondence:** Julija Armalytė: julija.armalyte@gf.vu.lt

| 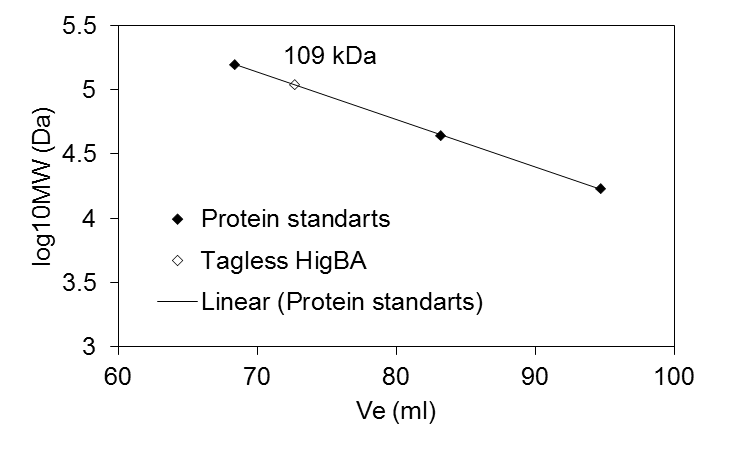 |
| --- |
| **Figure S4.** Size-exclusion chromatography of tag-less HigBA2_Ab_ protein complex. The proteins used for molecular mass standard curve were γ-globulin (158 kDa), ovalbumin (43 kDa) and myoglobin (17 kDa) (Bio-Rad), indicated as black diamonds, empty diamond indicates the position of HigBA2_Ab_ protein complex calculated size. |
